# Predicting severity of acute appendicitis with machine learning methods: a simple and promising approach for clinicians

**DOI:** 10.1186/s12873-024-01023-9

**Published:** 2024-06-18

**Authors:** Hilmi Yazici, Onur Ugurlu, Yesim Aygul, Mehmet Alperen Ugur, Yigit Kaan Sen, Mehmet Yildirim

**Affiliations:** 1 General Surgery Department, Marmara University Pendik Research and Training Hospital, Istanbul, Turkey; 2 Faculty of Engineering and Architecture, Izmir Bakircay University, Izmir, Turkey; 3 Department of Mathematics, Ege University, Izmir, Turkey; 4 General Surgery Department, University of Health Sciences Izmir Bozyaka Research and Training Hospital, Izmir, Turkey

**Keywords:** Appendicitis, Machine learning, Prediction, Severity

## Abstract

**Backgrounds:**

Acute Appendicitis (AA) is one of the most common surgical emergencies worldwide. This study aims to investigate the predictive performances of 6 different Machine Learning (ML) algorithms for simple and complicated AA.

**Methods:**

Data regarding operated AA patients between 2012 and 2022 were analyzed retrospectively. Based on operative findings, patients were evaluated under two groups: perforated AA and none-perforated AA. The features that showed statistical significance (*p* < 0.05) in both univariate and multivariate analysis were included in the prediction models as input features. Five different error metrics and the area under the receiver operating characteristic curve (AUC) were used for model comparison.

**Results:**

A total number of 1132 patients were included in the study. Patients were divided into training (932 samples), testing (100 samples), and validation (100 samples) sets. Age, gender, neutrophil count, lymphocyte count, Neutrophil to Lymphocyte ratio, total bilirubin, C-Reactive Protein (CRP), Appendix Diameter, and PeriAppendicular Liquid Collection (PALC) were significantly different between the two groups. In the multivariate analysis, age, CRP, and PALC continued to show a significant difference in the perforated AA group. According to univariate and multivariate analysis, two data sets were used in the prediction model. K-Nearest Neighbors and Logistic Regression algorithms achieved the best prediction performance in the validation group with an accuracy of 96%.

**Conclusion:**

The results showed that using only three input features (age, CRP, and PALC), the severity of AA can be predicted with high accuracy. The developed prediction model can be useful in clinical practice.

## Highlights

ML models can be used in all parts of medical treatments.

With good features, it would be useful in the prediction of surgical pathologies.

ML models are strong predictors of the severity of acute appendicitis.

With simple and easily found tools, the Logistic Regression algorithm predicted the severity of acute appendicitis with 96% accuracy.

This study also used an unseen data set to validate the results of training data. This increased the reliability of the prediction models.

## Introduction

Acute appendicitis (AA) is one of the most common pathologies among all emergent surgical procedures. The lifetime incidence is 7% in the whole population [1]. A clinical diagnosis can be made with clinical symptom assessment, a physical examination, laboratory tests, and radiological imaging in patients admitted to the emergency department. In recent years, easily accessible methods, such as C-reactive protein (CRP), leukocyte count, neutrophil to lymphocyte ratio (NLR), total bilirubin, multislice computed tomography, and ultrasonography imaging techniques have been used in the diagnosis of AA [2, 3]. Clinical symptoms can vary in many patients [4, 5]; hence, specific instruments were developed for the diagnosis. The most popular of these instruments is the Alvarado Scoring System, which was developed by Alfredo Alvarado in 1985 [6]. The Appendicitis Inflammatory Response Score was developed by Andersson et al., who combined the Alvarado with CRP and accurate prior results in diagnosing AA [7]. The purpose of all these systems is the early detection of AA and the avoidance of negative appendectomies and laparotomy-laparoscopies. In addition, delay in the diagnosis of AA may lead to complications [8, 9]. Due to the high incidence rate of acute appendicitis, the necessary tests should be available and easily performed at almost every hospital to help diagnose the disease without delay. Distinguishing simple and perforated/complicated cases when conducting these basic examinations is crucial in planning treatment and referring these cases to experienced centers.

Artificial Intelligence is a subfield of computer science and engineering that seeks to develop intelligent systems that can simulate human-like cognitive abilities. Machine Learning (ML) is a subfield of Artificial Intelligence that focuses on developing algorithms and statistical models that enable computers to learn from experience and improve without explicit programming autonomously. In the field of healthcare, ML algorithms analyze medical records and imaging data to support disease diagnosis, treatment planning, and drug discovery. In addition, ML provides crucial solutions for precision medicine, which endeavors to provide specific medical treatments to individual patients based on their unique genetic history, lifestyle, and environment. In several fields of medicine, ML has been used to help clinicians in diagnosis, treatment, and various modalities [10–14]. ML algorithms were also adopted in many studies to diagnose and treat AA [15, 16]. In addition to these studies, several researchers used ML algorithms to predict and diagnose different diseases with similar workflows [17–19].

This study evaluated the clinicopathological characteristics of AA patients in a tertiary center and investigated the predictive performances of the different ML algorithms. The primary objective of this research is to develop a simple and reliable prediction model using preoperative data to support physicians in assessing operative outcomes.

## Methods

Data from patients diagnosed with AA who underwent an emergency appendectomy between 2012 and 2022 in the hospital’s general surgery department were analyzed retrospectively. Patients under age 18 and those who had undergone an elective appendectomy for various reasons were excluded from the study. Patients with missing data were also excluded. An operational decision was made if the following were present: classic symptoms include right lower quadrant abdominal pain, tenderness at McBurney’s point, fever, nausea, vomiting, and elevated white blood cell count. Imaging confirmation (ultrasound or CT scan) shows signs of appendicitis, suspicion of complications, and failure of Non-Operative Management. Patients were examined within two groups: perforated AA and Non-Perforated AA. The diagnosis of perforated AA was defined according to the surgeon’s operative findings.

Demographics, including age and gender, peripheral blood analysis such as white blood cell (WBC) count, neutrophil count, lymphocyte count, platelet count and NLR, total bilirubin (TB), and CRP were recorded. The laboratory parameters chosen in the study were based on those that can be easily determined in almost all emergency departments. Multislice abdominal computerized tomography and abdominal ultrasound were used for preoperative radiologic evaluation. Appendix diameter (AppD) was calculated from preoperative radiologic images. The presence or absence of Periapendiculer Liquid Collection (PALC) in the radiological images was noted. Postoperative complications were evaluated according to the Clavien-Dindo classification [20].

### Statistical analysis

SPSS version 24.0 (Spss inc. IBM, Chicago, US) was used for statistical analysis. The clinical and laboratory features were compared between the perforated AA and none-perforated AA groups. Data on quantitative variables are presented as the median (minimum-maximum) and frequencies for qualitative variables. A Chi-square [21] test or Fisher’s exact test [22] was used for the nominal variable and the Mann-Whitney U test for the continuous variable with abnormal distributions. Univariate logistic analysis was used for feature selection. Multivariate logistic regression analysis was used to determine the significant features of the univariate analysis.

## Machine learning algorithms

In this study, six different ML algorithms: k-Nearest Neighbor (k-NN), Decision Tree (DT), Logistic Regression (LR), Support Vector Machine (SVM), Gaussian Naïve Bayes (GNB), and Multi-Layer Perceptron (MLP) were used for predicting simple and complicated AA.

k-NN is a supervised ML algorithm for classification and regression. k-NN works by finding the k nearest training instances to a given test instance and using those neighbors to make a prediction. k-NN is a simple yet powerful algorithm based on the idea that similar instances are likely to have the same class label [23].

DT is a supervised ML algorithm for classification and regression. DT works by recursively splitting the data into subsets based on the values of the input features and making predictions based on the majority class in each subset. DTs are simple to interpret, visualize, and implement and are commonly used for feature selection, outlier detection, and handling non-linear and complex relationships between features and targets [24].

LR is a supervised ML algorithm for binary classification. LR works by modeling the relationship between the input features and the probability of the positive class and using that model to make predictions. LR is a simple and interpretable algorithm widely used for binary classification problems and is often used as a baseline for comparison with more complex models [25].

SVM is a supervised ML algorithm for classification and regression. SVM works by finding the hyperplane that maximally separates the data into two classes and using that hyperplane to make predictions. SVM is a robust algorithm that is particularly well-suited for problems with high-dimensional data and problems with many features relative to the number of instances [26].

MLP is a supervised ML algorithm for classification and regression. MLP uses a feedforward neural network with one or more hidden layers to model the relationship between the input features and the target [27].

GNB is a supervised ML algorithm for classification. GNB makes predictions based on the Bayes theorem, which states that the probability of a class given the features is proportional to the prior probability of the class and the conditional probability of the features given the class [28].

**Fig. 1:**
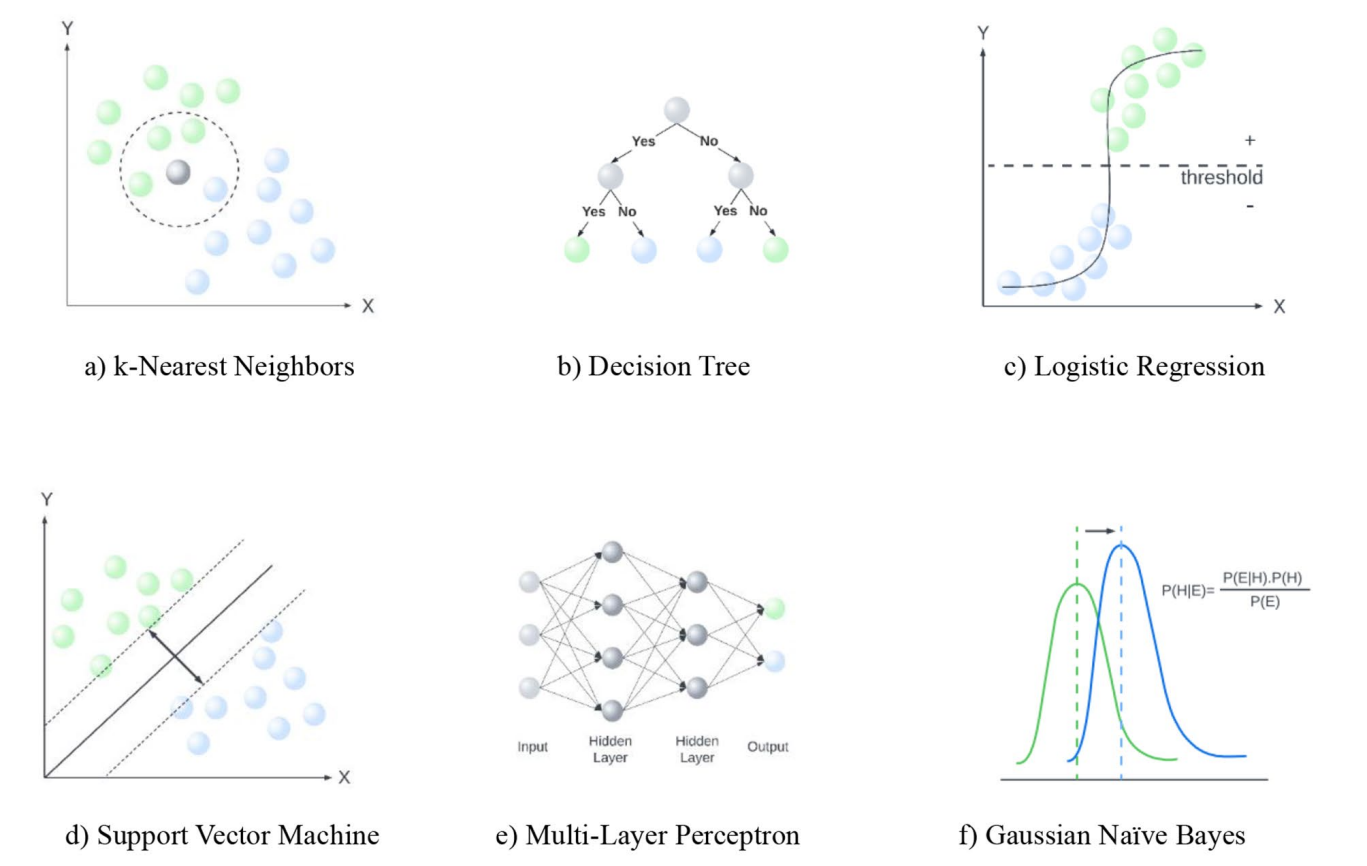
Comparison of k-NN, DT, LR, SVM, MLP, and GNB: Patients with perforations are represented by green circles, non-perforations by blue circles, and unclassified patients by grey circles

Figure 1 gives conceptual illustrations of the applied ML algorithms. These algorithms were carried out with Scikit-learn, one of the well-known Python libraries for ML.

Determining the optimal hyperparameters is a crucial aspect of enhancing the prediction performance of ML algorithms. In this study, we utilized a grid search technique within the Scikit-learn framework to find the best set of hyperparameters. This method searches through a range of predefined parameters and provides the ones with the highest prediction accuracy. Table 1 gives the interval of the hyperparameters for each ML algorithm used in the grid search.

**Table 1:** Grid-Search parameters of ML algorithms

TablesAlgorithms	Parameters
K-NN	n_neighbors = {1, …, 20}, metric = {minkowski, euclidean, manhattan}
DT	max_depth = {3, …, 8}, criterion = {gini, entropy}
LR	C = {0.01, 0.012, 0.013, …, 10,000}, penalty = {l1, l2, elasticnet}, solver = {newton-cg, lbfgs, liblinear}
SVM	C = {0, 0.1, 0.2, …, 1.9}, gamma = {scale, auto}, kernel = {linear, poly, rbf, sigmoid}
MLP	hidden_layer_sizes = {1, …, 20}, max_iter = {1000, 1500, 2000}, solver = {lbfgs, sgd, adam}
GNB	var_smoothing = {0.01, 0.011, 0.012, …, 100}

The prediction performance of the ML algorithms is assessed using several metrics such as Accuracy, Sensitivity, Specificity, Positive Predictive Value (PPV), and Negative Predictive Value (NPV). Accuracy is the proportion of correct predictions made by the model. Sensitivity (Recall or True Positive Rate) measures how many models correctly identified positive cases. Specificity is a performance metric in binary classification problems that measures the proportion of negative instances correctly identified as negative by the classifier. PPV is defined as the proportion of positive predictions that are actually correct. NPV is defined as the proportion of negative predictions that are actually correct. In addition, we performed a receiver operating characteristic (ROC) curve analysis for these algorithms and compared the AUC values.

This study was approved by the Ethics Committee of the University of Health Sciences Izmir Bozyaka Training and Research Hospital (decision date: 01.12.2022 no: 2022 / 163). All methods were performed in accordance with the relevant guidelines and regulations.

## Results

Between January 2012 and December 2022, 1568 patients underwent appendiceal surgeries. A total number of 256 patients were excluded because of missing data. Among them, 128 were excluded from the study because they were under 18 years old. Fifty-two patients who underwent elective appendectomies due to other reasons (i.e., mucinous appendiceal disease combined with gynecological pathologies and plastron appendicitis, etc.) were also excluded. Overall, 1132 patients who underwent appendectomy fulfilled the inclusion criteria for this study. The median age of the entire cohort was 37 (IQR: 27–50), and the majority was male [n: 847 (74%)]. There were 990 patients in the non-perforated AA group and 142 patients in the perforated AA group. Detailed demographic and baseline preoperative characteristics of the two groups are given in Table 2. With univariate analysis, the WBC count and platelet count were similar between the groups. However, the median age, gender, neutrophil count, lymphocyte count, NLR, TB, CRP, AppD, and PALC were significantly different between the two groups. A multivariate analysis of the features which was found to be significant in the univariate analysis was performed. In the multivariate analysis, age, CRP, and PALC continued to be independent factors for perforated AA (Table 3).

**Table 2:** Patient Demographics

*N* : 1132 Mean (± SE), Median(IQR)	Non-Perforated (*N*: 990)	Perforated (*N*: 142)	*p*
Age (Median)	35(27–48)	47(37–64)	**< 0.001**
Gender (%)			
Male Female	755 (76%) 235 (24%)	87 (61%) 55 (39%)	**< 0.001**
WBC (G/L)(Median)	13.4(10.8–16.4)	14.1 (11.2–17.4)	0.243
Neutrophil (G/l) (Median)	10.3 (7.9–13.1)	11.5(8.3–14.6)	**0.037**
Lymphocyte(G/l)(Median)	1.8(1.3–2.5)	1.5(0.9–2.1)	**0.002**
NLR (Median)	5.5(3.5–8.9)	7.4(4.6–13.6)	**< 0.001**
Platelet(µl)(Median)	247 (208–285)	269(200–307)	0.982
Total Bilirubin(Median) (mg/dL)	0.73(0.5–1.3)	1(0.6–1.5)	**< 0.001**
CRP (mg/L)(Median)	13.8(4.2–42.4)	130.3(40.7-204.6)	**< 0.001**
AppD (mm)(Median)	10(8.5–12)	12(10–15)	**< 0.001**
PALC			
Presence Absence	216 (22%) 774 (78%)	102 (72%) 40 (28%)	**< 0.001**

SD: Standard Error, IQR: InterQuartile Range, WBC: White Blood Cell, NLR: Neutrophil/Lymphocyte Ratio CRP: C-Reactive Protein, AppD: Appendix Diameter, PALC: Peri-Appendicular Liquid Collection (Significant Values are shown in bold.)

**Table 3:** Univariate and Multivariate Logistic Regression Analysis

*N* : 1132	HR	95% CI	*p*	HR	95% CI	*p*
Age	0.959	0.948–0.969	**< 0.001**	0.961	0.961–0.990	**0.001**
Gender	2.031	1.406–2.935	**< 0.001**	0.364	0.820–2.269	0.231
WBC	0.986	0.967–1.006	0.165			
Neutrophil	0.924	0.887–0.962	**< 0.001**	0.970	0.921–1.037	0.274
Lymphocyte	1.541	1.231–1.929	**< 0.001**	1.104	0.887–1.373	0.376
Platelet	0.965	0.812–1.023	0.680			
Total Bilirubin	0.837	0.751–0.954	**0.042**	1.004	0.993–1.045	0.464
CRP	0.985	0.982–0.987	**< 0.001**	0.988	0.985–0.990	**< 0.001**
AppD	0.981	0.942–0.996	**0.026**	0.989	0.976–1.002	0.107
PALC	9.399	6.324–13.967	**< 0.001**	6.623	4.167–10.527	**< 0.001**

NLR: Neutrophil/Lymphocyte Ratio CRP: C-Reactive Protein, AppD: Appendix Diameter, PALC: Peri-Appendicular Liquid Collection. (Significant Values are shown in bold.)

Postoperative complications, assessed according to the Clavien-Dindo Classification, are summarized in detail in Table 4. Although Grade IIIA complication rates were similar between the two groups, Grade I-II and Grade IIIB complications were significantly higher in the perforated AA group (p: <0.001 and p: 0.005, respectively). Total morbidities were also higher in the perforated AA group (p: <0.001).

**Table 4:** Perioperative Complications (30 days)

Total *N*: 1132	Non-Perforated (*N*: 990)	Perforated (*N*: 142)	*p*
Complication ≥ Grade III Intra-Abdominal Abscess Ileus Stump Leakage Wound Infection (Grade IIIA) Fascial Dehiscence Iatrogenic Colon Perforation	[N:12 (1%)] 6 0 1 3 1 1	[N: 6 (4%)] 3 1 1 0 1 0	0.058 **0.008** 0.109 0.511 0.705 0.705
Complication Grades*			
I-II IIIA IIIB Total	34 9 3 46(5%)	28 3 3 34(24%)	**< 0.001** 0.190 **0.005** **< 0.001**

* Grades according to the Clavian-Dindo Classification (Significant Values are shown in bold.)

We developed a prediction model based on different preoperative data sets to predict simple and complicated AA. The first data set (data set 1) includes Age, Gender, neutrophil and lymphocyte count, NLR, TB, CRP, AppD, and PALC, which were significant in the univariate analysis as input parameters/features, whereas the second data set (data set 2) only included Age, CRP, and PALC, which were found to be independent factors for perforation in the multivariate analysis. Each data set consisted of 1132 samples, and the output parameter was perforated AA in both data sets.

In supervised ML algorithms, the data set is generally split into three sets: the training data, the test data, and the validation data. The training data was used to train the model and learn the relationships between the inputs and outputs, while the test data was used to evaluate the model’s performance. The validation data helped us to determine how well the prediction model would perform on unseen data. In this study, both data sets (data set 1 and data set 2) were randomly split into three parts: 932 samples for training, 100 samples for testing, and 100 samples for validating.

The 10-fold cross-validation method was used to evaluate the performance of all ML algorithms more accurately. The optimal hyperparameters of the ML algorithms found by the grid search for each data set are given in Table 5.

**Table 5:** The optimal parameters of the ML algorithms

Algorithms	Data set 1	Data set 2
k-NN	n_neighbors = 13, metric = minkowski	n_neighbors = 19, metric = manhattan
DT	max_depth = 4, criterion = gini	max_depth = 3, criterion = gini
LR	C = 4.037, penalty = l1, solver = liblinear	C = 0.498, penalty = l2, solver = newton-cg
SVM	C = 1.0, gamma = scale, kernel = poly	C = 0.1, gamma = scale, kernel = poly
MLP	hidden_layer_sizes = 2, max_iter = 1000, solver = lbfgs	hidden_layer_sizes = 16, max_iter = 1000, solver = lbfgs
GNB	var_smoothing = 0.04	var_smoothing = 0.196

**Fig. 2:**
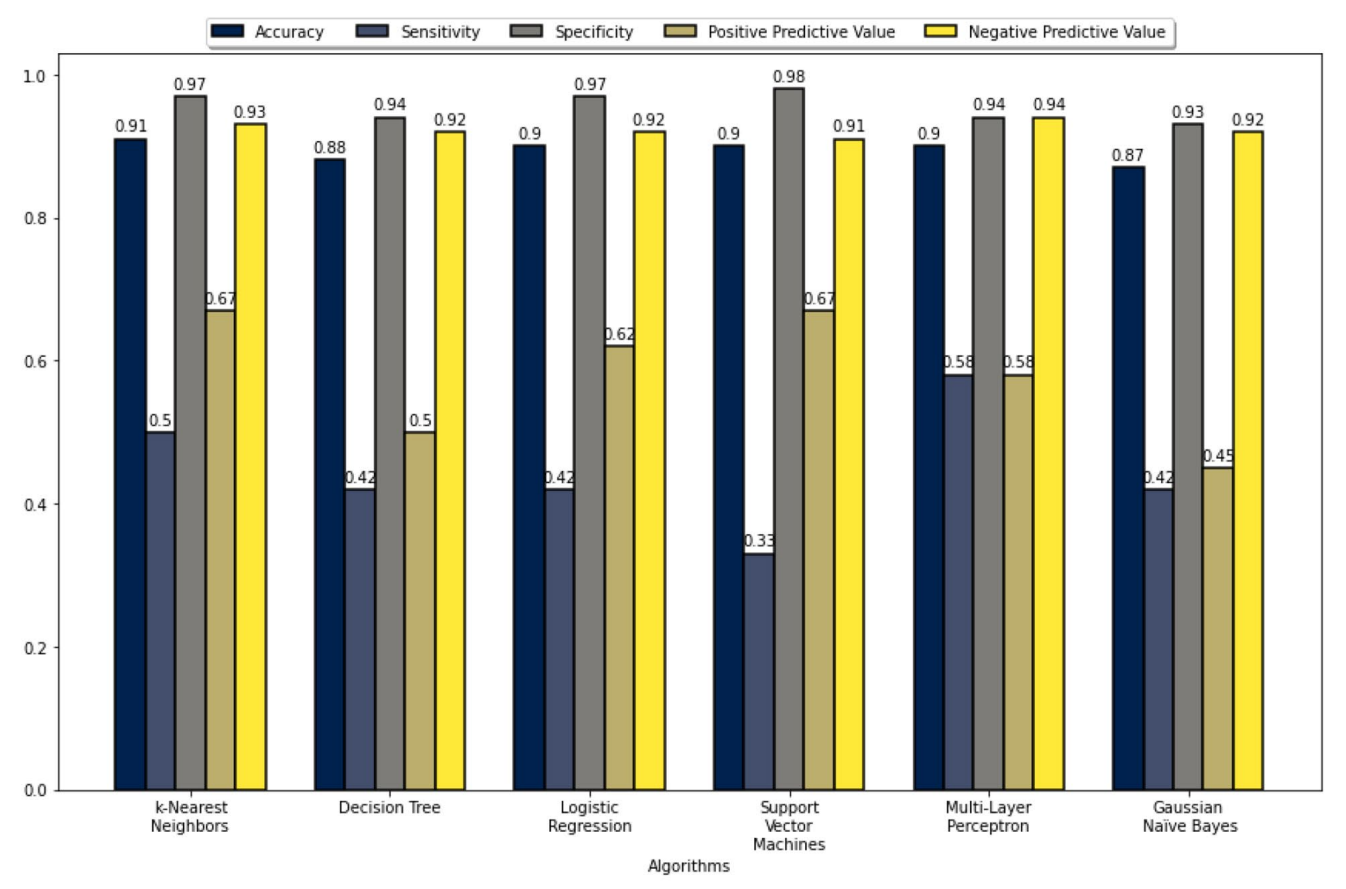
Performance evaluation metrics of the ML algorithms on test data 1

**Fig. 3:**
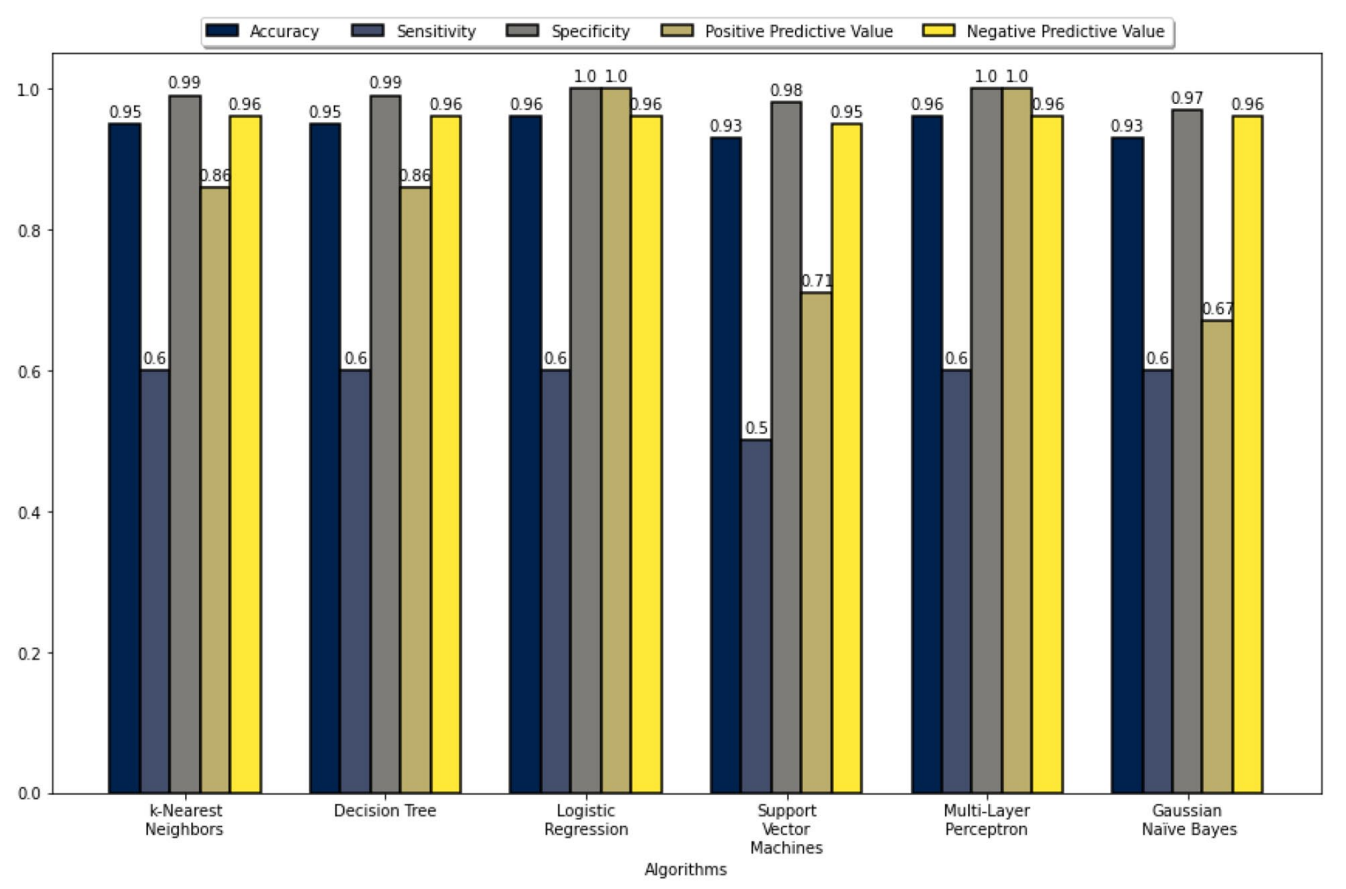
Performance evaluation metrics of the ML algorithms on validation data 1

**Fig. 4:**
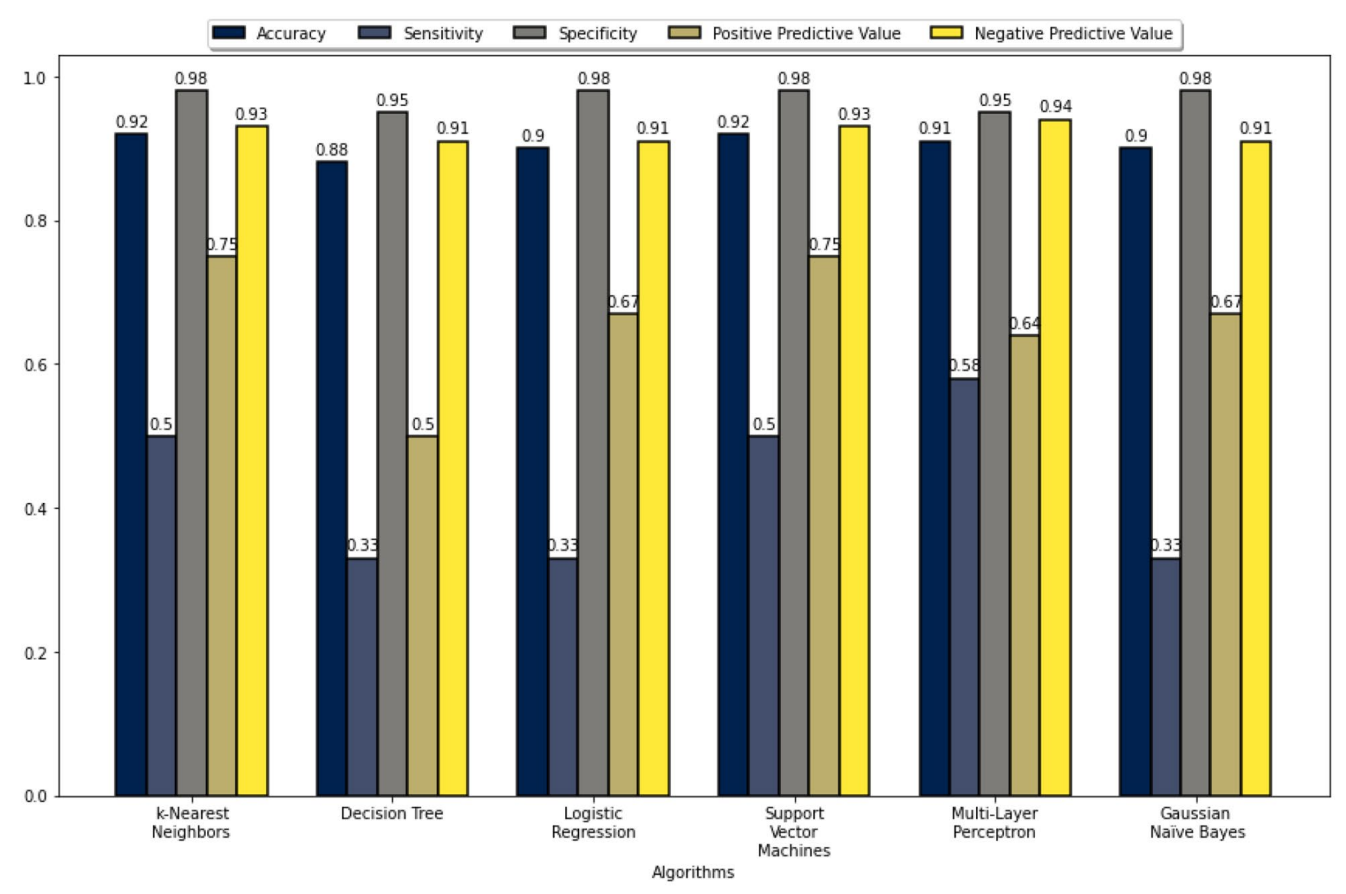
Performance evaluation metrics of the ML algorithms on test data 2

**Fig. 5:**
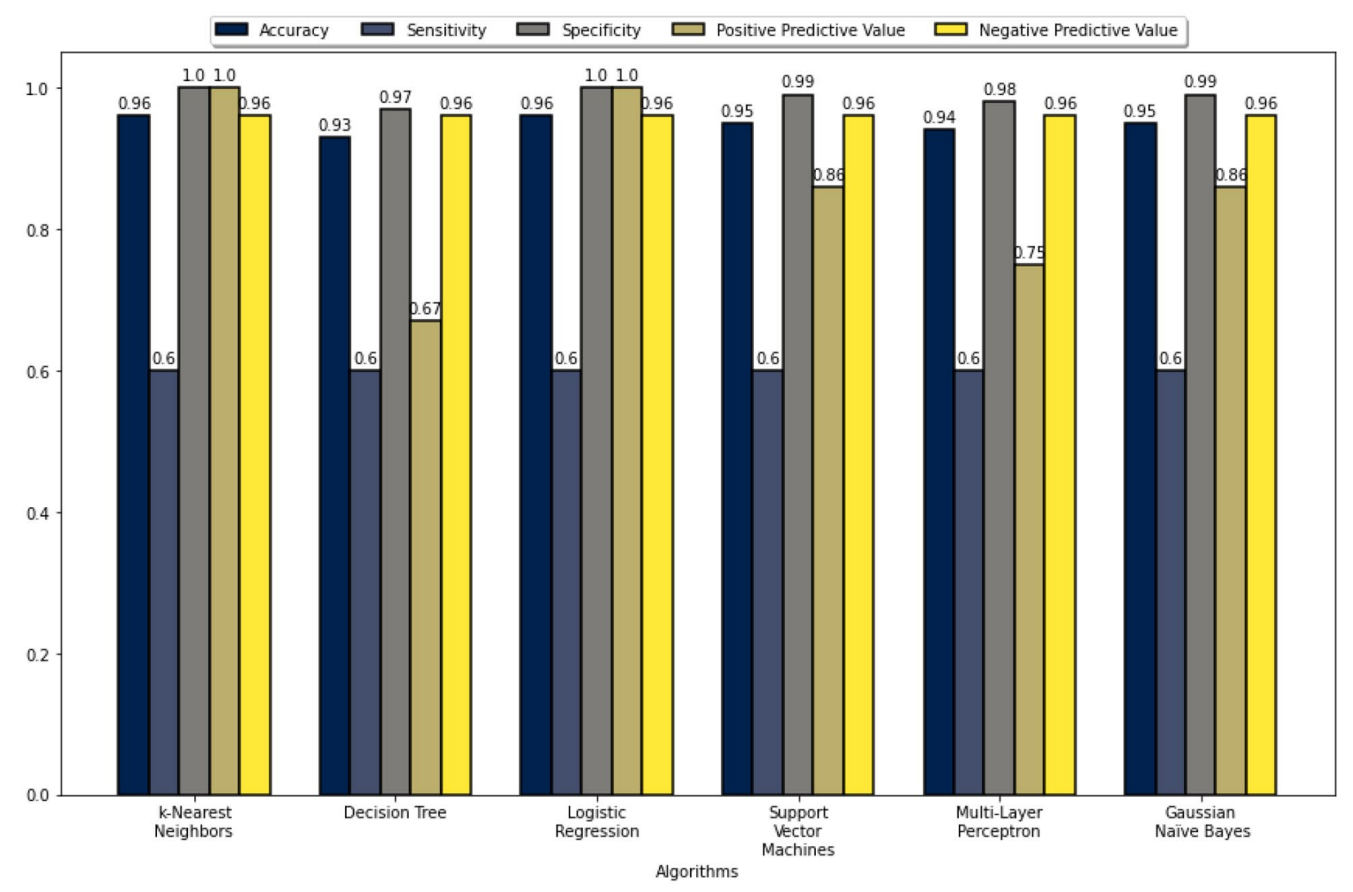
Performance evaluation metrics of the ML algorithms on validation data 2

The five performance measures for all the ML algorithms’ accuracy, sensitivity, specificity, PPV, and NPV are summarized in Figs. 2, 3, 4, and 5. These figures show that the k-NN, LR, SVM, and MLP algorithms demonstrate high performance, with prediction accuracies exceeding 90% on the test data for each data set. In addition, the results indicate that these algorithms performed more efficiently on data set 2. These figures also show that all algorithms achieved a prediction accuracy of over 93% on the validation data for each data set. Figure 3 demonstrates that the LR and MLP algorithms accurately classified 90 out of 100 samples as “Non-Perforated” for data set 1. Furthermore, it seems that all these algorithms, except for SVM, correctly classified six unseen samples as “Perforated” for data set 1. Considering Figs. 2 and 3, the LR algorithm has the best prediction performance on data set 1, with 96% accuracy, 60% sensitivity, 100% specificity, 100% PPV, and 96% NPV. Figure 5 indicates that the k-NN and LR algorithms accurately classified 90 of the 100 data samples as “non-Perforated”. However, four data samples were misclassified by the k-NN and LR algorithms. Similarly, all algorithms appear to classify six previously unseen samples as “Perforated” accurately. Considering Figs. 4 and 5, the k-NN and LR algorithms showed the highest prediction performance with an accuracy of 96%. Consequently, the LR algorithm was found to have the highest accuracy in classifying the unseen samples as Perforated and Non-Perforated compared to the other algorithms.

**Fig. 6:**
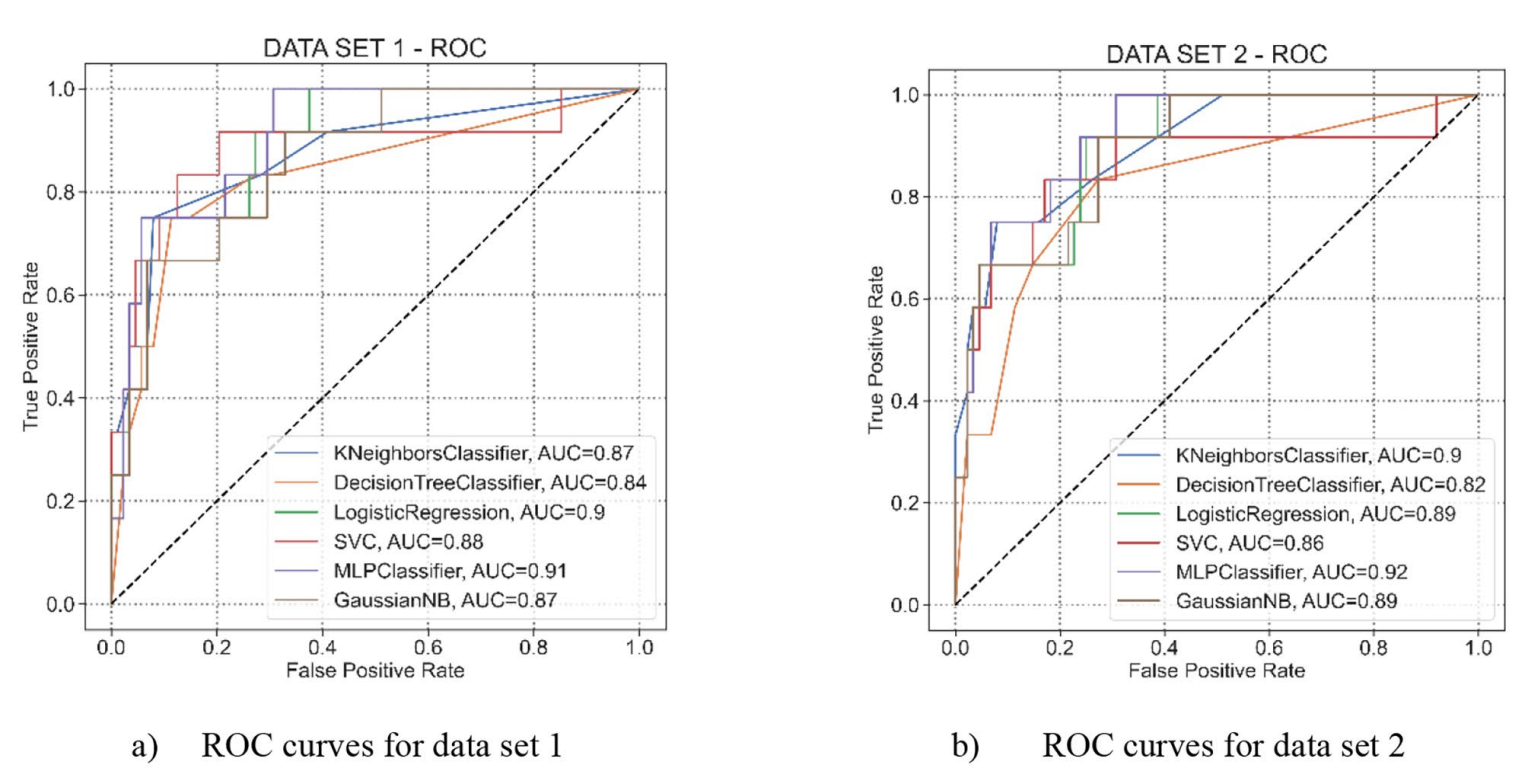
Comparison of the ROC curves of ML algorithms. The x-axis displays the false-positive rates (1-specificity) while the y-axis displays the true-positive rates (sensitivity)

Figure 6 shows the AUC curve for the ML algorithms on data sets 1 (Fig. 6a) and 2 (Fig. 6b). In Fig. 6a, the AUC values of all algorithms are above 84%. However, using only Age, CRP, and PALC as the input parameters increased the AUC values of the k-NN, MLP, and GNB algorithms to 90%, 92%, and 89%, respectively.

## Discussion

Our research has two novel contributions to the related literature. The first one is using the validation data set, which tests the prediction model on the unseen samples. To the best of our knowledge, this is the first study that uses a validation data set to predict the severity of AA. Hence, our results are more reliable than those of the previous works. The second main contribution of this study is our investigation of the effect of the input features on the performance of the ML algorithms. Again, to the best of our knowledge, this is the first study evaluating ML models for diagnosing the severity of AA in adults on two different preoperative data sets and investigating the effect of input features using two different data sets. This study showed that multidisciplinary approaches with clinicians and data scientists might help improve an ML model that accurately predicts critical health conditions. Clinicians can determine the necessary input features both with clinic decisions and statistically, while data scientists can develop the best ML model for this clinic condition. The study aimed to test the input features that can be found at all hospital levels for the detection of complicated AA in ML models. Moreover, models developed with age, CRP, and PALC input features, along with clinician suggestions, also obtained similar results. As the number of similar studies increases and other researchers demonstrate the reliability of ML on different data sets for AA, such studies can be used in healthcare centers with easy-to-use tools.

Many studies showed a strong relationship between peripheral blood analysis findings and AA [29, 30]. In the present study, there was no significant difference between the WBC and platelet counts between the two groups. However, there was a significant difference in terms of neutrophil count, lymphocyte count, and NLR between the perforated AA group and the non-perforated AA group. CRP level is known to be a significant marker for almost all inflammatory processes. Moreover, it is an important marker for diagnosing AA [31]. In this study, CRP level also showed a significant relation with perforated AA, and this is consistent with the current studies. TB levels were higher in the Perforated AA group than in the Non-perforated group, which is supported by the current studies [32, 33]. Several studies have shown that older age is a risk factor for more complicated AA [34, 35]. Comorbid diseases become more common with growing age. On the contrary, physiological reserves decrease significantly with increasing age. These might lead to complications becoming more severe. This study also found that age was an independent factor for complicated AA. Thus, scoring systems or ML models should include age as a factor during the decision-making process.

Many diagnostic tools have been developed for diagnosing AA [36–39]. Still, the predictive performances of these scores are controversial. Deiters et al. conducted a study on 216 elderly AA patients regarding the usefulness of the Alvarado score in predicting the severity of AA. They reported that The Alvarado score did not differ in both groups preoperatively [37]. Haak et al. argued that both AIR and Alvarado scores have limited capacity to distinguish simple and complicated AA [36]. They also found 0.670 and 0.598 AUC values, respectively. In the present study, every ML model determined AUC values more than 0.84. The results indicated the superiority of ML models over Alvarado and AIR scores predicting complicated AA. Atema et al. have devised two Scoring systems for Appendicitis Severity (SAS) that integrate radiological findings with clinical and biochemical characteristics: one based on US features (SAS-US) and the other based on CT features (SAS-CT) [38]. Sensitivity, specificity, PPV, and NPV for US-SAS are 97%, 46%, 42%, and 97%, respectively. For the scoring system with CT features, SAS-CT, these test features are 90% sensitivity, 70% specificity, 55% PPV, and 95% NPV. The SAS scoring systems have remarkable diagnostic assets, notably high sensitivity and negative predictive value, for excluding complicated AA. However, scores do not demonstrate strong performance in confirming complicated AA. This result also emphasizes the importance of radiological findings in diagnosing perforated-complicated AA. Moreover, both Alvarado and AIR scores contain physical examination points in the total score. Hence, This makes both scoring systems clinician-dependent tools. Therefore, this study aims to develop ML tools with certain features that might lead to eliminating this condition and obtaining more standardized results.

It is well-known that findings in ultrasound (US), computerized tomography (CT), and magnetic resonance are all important for diagnosing AA. While magnetic resonance evaluation is neither common nor useful, especially in rural areas, CT and the US are applicable in almost every hospital. Hence, we analyzed CT and US reports for AppD and PALC. Recent studies showed that AppD is important for both simple AA [40] and complicated AA [41] diagnoses. The median AppD was significantly higher in the perforated AA group, which is consistent with recent studies. PALC was observed significantly more often in the perforated AA group, and this is also supported by the existing literature [40, 42].

Despite advances in surgical techniques and medical treatments in recent years, complicated appendicitis is still a challenge to surgeons. Recently, several studies have investigated nonoperative approaches to AA treatment [43, 44]. However, AA surgery still has substantial complication rates even for a relatively simpler surgical approach. In a large cohort study by Sood et al. from the American College of Surgeons National Surgical Database, the Grade III-V complication rate in AA surgery was between 2.5 and 5% [45]. The present study had 18 (1.6%) grade IIIA-IIIB complications, and no perioperative mortality was observed. Although the complication rates were lower than in existing studies, complications in the perforated AA group were still higher than those not perforated in this study. This also supports that preoperative diagnosis of more complicated cases might be essential for the treatment of AA.

In recent years, many researchers have used ML algorithms to predict AA. Hsieh et al. employed different ML algorithms such as SVM, LR, Random Forest, and Artificial Neural Networks to diagnose acute appendicitis using 16 input features [46]. They reported AUC values ranging from 77 to 98%. Nevertheless, it is crucial to acknowledge that the limited sample size of 180 individuals reduces the reliability of their results.

Park et al. used SVM to diagnose acute appendicitis [47]. Their data set consists of 760 samples with 10 different input features. The study reported that the AUC values ranged from 62.1 to 99.7%. However, although ML showed satisfying results in diagnosing AA in their cohort, predicting perforated or complicated cases remained controversial.

Akmese et al. studied data from the records of 595 patients for the diagnosis of acute appendicitis [48]. The authors used Neural Networks, k–NN, LR, SVM, Random Forest, and Gradient Boosting Tree. According to the study, the Gradient Boosted Tree algorithm showed the highest prediction performance at an accuracy of 95%. However, the absence of AUC values raises concerns regarding the performance of the models.

Mijwil et al. conducted a study using data from the records of 625 patients to diagnose acute appendicitis [49]. To predict AA, they compare several ML algorithms, including LR, SVM, DT, Naive Bayes, Generalized Linear Model, Gradient Boosted Tree, and Random Forest. The findings of the study show that the accuracy of the algorithms ranges from 64.74 to 83.75%. Nevertheless, similar to the study of Akmese et al. [48], the authors did not report AUC values.

The majority of recent studies have examined ML in the diagnosis of AA [46–49]. However, this study mainly focused on predicting simple and complicated AA preoperatively. In this manner, we investigated the performance of the different ML algorithms for predicting simple and complicated AA. One of the strengths of this study is that the size of the data set is larger than the previous ones. These properties allow for a more comprehensive analysis and a better evaluation of the ML algorithms.

This study also has some limitations. First, the retrospective design might lead to selection or analytic biases. Second, the study cohort was from a single center, which could have led to sample homogeneity. Thus, prospective multicenter studies are needed to correct this potential issue. Finally, the cases that managed non-operatively were missing in this study. This might have affected the results.

## Conclusion

The use of technological developments in clinical practice is essential in spending less time and convenience for healthcare professionals. ML algorithms are an actual and developing topic of technological development. Over time, it will continue to gain more space in medical sciences. This study showed that the ML algorithm could achieve high predictive performance for diagnosing simple and complicated AA using only a few input features. Therefore, it should be discussed in a further large series.

## Data Availability

The data sets used and/or analyzed during the current study are available from the corresponding author upon reasonable request.
